# Dementia Risk Scores and Their Role in the Implementation of Risk Reduction Guidelines

**DOI:** 10.3389/fneur.2021.765454

**Published:** 2022-01-04

**Authors:** Kaarin J. Anstey, Lidan Zheng, Ruth Peters, Scherazad Kootar, Mariagnese Barbera, Ruth Stephen, Tarun Dua, Neerja Chowdhary, Alina Solomon, Miia Kivipelto

**Affiliations:** ^1^School of Psychology, University of New South Wales, Sydney, NSW, Australia; ^2^Neuroscience Research Australia, Randwick, NSW, Australia; ^3^Department of Neurology, Institute of Clinical Medicine, University of Eastern Finland, Kuopio, Finland; ^4^The Ageing Epidemiology Research Unit, School of Public Health, Imperial College London, London, United Kingdom; ^5^Brain Health Unit, Department of Mental Health and Substance Use, World Health Organization, Geneva, Switzerland; ^6^Division of Clinical Geriatrics, Department of Neurobiology, Center for Alzheimer's Research, Care Sciences and Society, Karolinska Institute, Stockholm, Sweden; ^7^Theme Inflammation and Aging, Karolinska University Hospital, Stockholm, Sweden; ^8^Institute of Public Health and Clinical Nutrition, University of Eastern Finland, Kuopio, Finland

**Keywords:** dementia, risk assessment, risk score, cognitive decline, risk factors

## Abstract

Dementia prevention is a global health priority. In 2019, the World Health Organisation published its first evidence-based guidelines on dementia risk reduction. We are now at the stage where we need effective tools and resources to assess dementia risk and implement these guidelines into policy and practice. In this paper we review dementia risk scores as a means to facilitate this process. Specifically, we (a) discuss the rationale for dementia risk assessment, (b) outline some conceptual and methodological issues to consider when reviewing risk scores, (c) evaluate some dementia risk scores that are currently in use, and (d) provide some comments about future directions. A dementia risk score is a weighted composite of risk factors that reflects the likelihood of an individual developing dementia. In general, dementia risks scores have a wide range of implementations and benefits including providing early identification of individuals at high risk, improving risk perception for patients and physicians, and helping health professionals recommend targeted interventions to improve lifestyle habits to decrease dementia risk. A number of risk scores for dementia have been published, and some are widely used in research and clinical trials e.g., CAIDE, ANU-ADRI, and LIBRA. However, there are some methodological concerns and limitations associated with the use of these risk scores and more research is needed to increase their effectiveness and applicability. Overall, we conclude that, while further refinement of risk scores is underway, there is adequate evidence to use these assessments to implement guidelines on dementia risk reduction.

## Introduction

The publication of the WHO guidelines on risk reduction of cognitive decline and dementia (1) is the first step to support the implementation of the action area on dementia risk reduction in the Global Action Plan on the Public Health Response to Dementia 2017–2025 (2). The guidelines signal that the observational and trial evidence base is sufficient to support translation of the research findings into policy and practice, but effective tools are needed for this purpose. Implementation of guidelines requires an infrastructure that is adaptable to individual settings within countries, health care systems, and communities (3), including the development of tools and resources on dementia risk reduction, and validated means of assessing risk factors. The use of such instruments can be informative both at the individual patient level, as well as at the health policy and planning level. In this article, we provide an overview of the key perspectives on dementia risk scores as assessment tools in the context of public health based on expert opinion regarding evidence-based research and practice. The following sections include (a) the rationale for dementia risk assessment, (b) methodological issues to consider when reviewing risk scores, (c) examples of dementia risk scores that are currently in use and their strengths and limitations, and (d) some comments on moving evidence into practice.

## Rationale for Risk Assessment

### Risk Assessment and Risk Scores for Chronic Diseases

A risk score is a weighted composite of risk factors that reflects the likelihood of developing a certain disease/condition/disorder. There is a long history of scores used in clinical practice to identify those at risk and for targeting primary prevention treatments accordingly (4). The most well-known example is probably the Framingham Risk Score (FRS), originally developed for the prediction of coronary heart disease (CHD) risk in adults (5). The original Framingham risk score combined both modifiable and non-modifiable risk factors, i.e., age, type 2 diabetes, smoking, blood pressure and total and high-density lipoprotein (HDL) cholesterol level, in a sex specific weighted total. This translated to a score in the form of a 10-year percentage increased risk of CHD. The components of the FRS have been supplemented and extended and reappear in multiple subsequent risk scores for cerebrovascular events, peripheral artery disease, and heart failure. These subsequent risk scores tend to involve varying combinations of risk factors, weighting algorithms, time periods, and validations across different populations (6). The use of scores for risk prediction has also extended to other cardiovascular disease areas, examples include prediction scores for intermittent claudication, fatty liver disease, type 2 diabetes, and, recently, dementia (6–8).

Risk scores can improve the identification of those at risk over and above the use of clinical judgement alone (4, 9, 10). When risk scores are converted into tools for use in practice, they may also facilitate communication of risk to patients and increase risk reduction treatments, in particular in the highest risk populations (9, 10). For example, cardiovascular risk charts using color coding allow easy assimilation of risk level by various combined categories including age, sex and risk factor status like blood pressure level, cholesterol level and smoking status (11, 12). These are recommended by professional and government organizations and are used to guide treatment decisions in primary prevention of cardiovascular diseases and diabetes (13–15). As health records are becoming more digitalised, there is also potential for semi-automated and dynamic risk scores that can not only take account of multiple risk factors, but add another aspect to risk prediction, that is, the variation in risk factor profile over time. This will be useful where trajectories of risk factor exposure vary over time and when their importance varies by age (16), as is the case with dementia.

### Dementia Risk Assessment and Risk Scores

A recent meta-analysis identified four types of risk scores for dementia that have been developed (17). These included four midlife risk models, 39 late-life risk models, 15 risk score models predicting progression from MCI to Alzheimer's disease (AD) and three risk models predicting risk of dementia in patients with diabetes. To our knowledge, although some existing vascular risk scores (e.g., FRS) have been used to predict dementia risk (18), a risk score designed to specifically predict vascular dementia risk has yet to be developed, which may indicate a gap in the research. The most basic scores use a binary scoring system (0 or 1 to correspond with the risk factor being present or absent), while other scores use beta weights from regression models. To date, all the dementia risk scores in the literature have preferred an additive approach. That is, individual weighted risk factors are summed (rather than multiplied or combined by some other function) to provide a total score. The weights attributed to risk score components have been derived either directly from analysis of cohort studies [e.g., (19)], or by combining effect sizes using meta-analytic techniques (20). The evidence base related to dementia risk factors continues to increase with publication of long-term follow-ups from well characterized, large, cohort studies (21). This will enable more complex algorithms to be developed that account for competing risk and time-ordering of risk factors. For example, risk scores could take into account competing risk of mortality from stroke or myocardial infarction (22), the correlation between risk factors occurring within an individual, or gender or ethnic differences in the weighting of specific component risk factors (23).

In contrast to the field of cardiovascular disease (3), dementia risk scores are not yet widely used in clinical settings. There may be a number of practical and methodological reasons why this is the case. For example, there is stigma and fear surrounding dementia embedded in community attitudes toward people with dementia (24) which may cause individuals to delay getting assessed or even seek appropriate diagnosis and care. Furthermore, dementia risk assessments do not drive treatment decisions for dementia *per se* but may indicate treatment for medical risk factors. The prodromal phase for dementia may last up to 30 years and the benefit of delivering prediction estimates is arguable. Rather, the focus on modifiable risk factors has a more tangible and immediate impact. Prediction over such long periods is complicated by the potential for change in the levels of different risk factors over time, and the impact of this on the overall risk of dementia. For example, an individual may have increased risk through rising blood pressure accompanied by reduced risk associated with weight loss. We consider some of the conceptual and methodological issues in more depth below.

## Conceptual and Methodological Considerations

Similar to other risk models, a dementia risk model in population-based or clinical settings needs to be checked for discriminative accuracy, predictive value, external validation, cost-effectiveness, and ethical implications (25). While a number of dementia risk scores have been developed, there are a variety of methodological issues that need to be considered when reviewing a risk score for dementia risk assessment.

### Target Age and Selection of Risk Factors for Inclusion in Risk Score Calculations

Risk scores for primary prevention of dementia generally target middle-aged adults and focus on modifiable risk factors that emerge during this time such as hypertension, high cholesterol and diabetes [e.g., CAIDE (26)]. However, other risk and protective factors that do not present with strong evidence in mid-life may still prove beneficial if identified early. For example, there is evidence for the protective effects of social and cognitive engagement, however this is largely drawn from studies of older adults (27) and it is unclear whether social and cognitive engagement should be included in risk assessment in midlife. Additionally, there is less evidence for physical activity and a healthy diet in mid-life reducing risk of dementia, but these health behaviors have wide benefits for related non-communicable diseases (NCDs) that are also risk factors for dementia (28). Therefore, while the benefits of these health behaviors are small over short term follow-up, they are likely to be far larger over decades. Considerations must also be made for the age at which to target risk scores for primary prevention. The definition of mid-life is typically 40 to 60 or 40 to 65 but may be younger in populations with shorter life expectancies or different life trajectories. For example, some indigenous communities define middle age as > 35 (29). It is possible that, as more evidence emerges, a wider age-span should be considered for primary prevention risk assessment (30–32).

### Inclusion of Non-modifiable Risk Factors

Consideration must also be given to the inclusion of non-modifiable risk factors for dementia in risk score calculations. Some of these may inform clinical management such as history of traumatic brain injury (TBI), family history of dementia (33), age and gender. If the evidence base is sufficient, an appropriate weighting and entry in the algorithm can be given to a non-modifiable risk factor [see (8) for an example]. However, at times, practicability must also be considered. For example polygenic risk scores (34, 35) and *APOE* genotype may improve the prediction of risk scores but are not widely available in clinical settings and the provision of genetic risk information may raise ethical issues or the need for genetic counseling.

Non-modifiable risk factors may interact with other modifiable risk factors, and provide critical information for tailoring of interventions to individuals, planning risk reduction strategies, and may guide allocation of resources at the population level. For example, individuals with history of TBI are also at increased risk of poorer mental health (36) which is in turn a risk factor for dementia, therefore knowledge of this risk profile may guide tailoring of health advice. Increasing understanding about how non-modifiable and modifiable risk factors interact may further inform clinical recommendations. This may also support increased motivation for individuals in the maintenance of protective behaviors once they are aware of their risk status. In the same vein, there may also be scope for the inclusion of social determinants of health into risk scores (e.g., socioeconomic status, occupation, social or economic adversities) as the evidence for these and their interactions with other risk factors begins to accumulate.

### Risk Scores for Secondary Prevention

Assessment of dementia risk may also occur in adults who already have symptoms of cognitive impairment or cognitive decline, or in whom there is established chronic disease that places individuals at known risk of dementia. Risk scores for dementia are already developed for populations with diabetes (37–39), but could also be developed for groups with common chronic conditions such as hypertension and stroke.

### Risk Score vs. Risk Indicator

Risk assessment in older populations may also include assessing “risk indicators” in addition to “risk factors.” Risk indicators are variables that directly indicate underlying changes in brain structure or function which are related to dementia. Examples of risk indicators include neuroimaging parameters such as white matter changes or atrophy [e.g., (40)], amyloid beta and phospho-tau, evidence of transient ischemic attacks and strokes, low cognitive screening scores, impaired Instrumental Activities of Daily Living (IADL), or memory or learning deficits. For low resource settings such as low to middle income countries (LMIC), or rural and remote locations, the use of a risk score is likely more economically viable than risk indicators that may require expensive tests or facilities that are not widely available.

### Distinguishing Risk Factor Research and Risk Reduction Research

Research into identification of risk factors, and measurement of risk factors needs to be distinguished from research and evidence on risk reduction. Risk factors are associated with increased risk in observational studies. They may or may not have a direct causal relationship with dementia. The plausibility of a causal association needs to be established through consideration of the body of scientific evidence that draws upon mechanistic animal studies, randomized controlled trials, as well as epidemiological studies with long follow-ups (41). It is possible that a genuine risk factor is identified, and can be assessed, but that reducing that risk factor does not in turn reduce the risk of dementia. The benefit of reversing risk factors needs to be established via randomized controlled trials and ideally the underlying biological mechanism by inclusion of biomarkers. The WHO Guidelines on Risk Reduction for Cognitive Decline and Dementia focused on interventions that reduce or reverse risk factors (1).

### Distinction Between a Risk Score and a Risk Tool for Use in Practice

**Figure 1:**
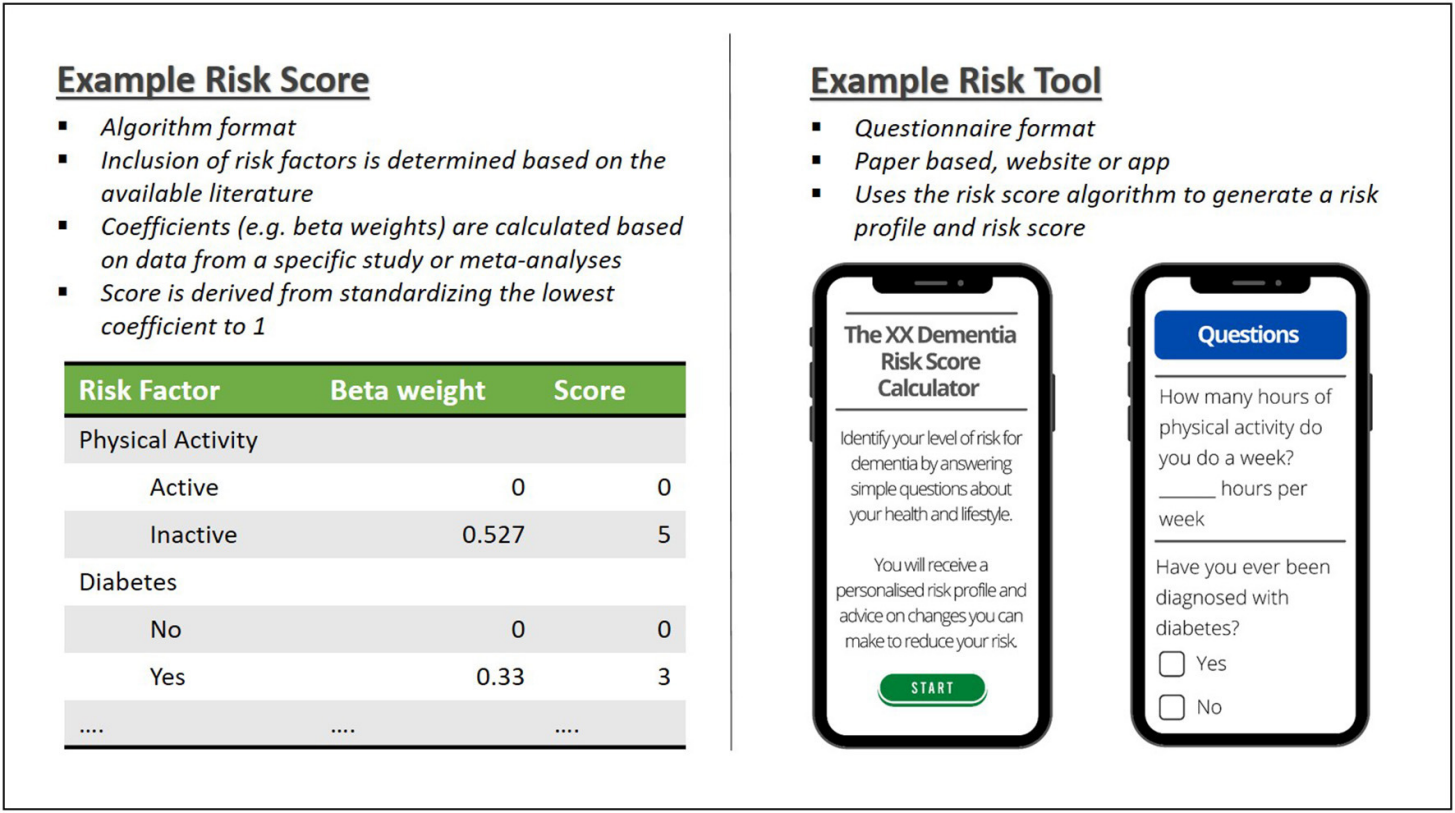
Risk score and risk tool examples.

A distinction can also be made between risk scores and a risk tool. Risk scores are statistical models derived from analysis of cohort studies that include a dementia outcome. While many authors have published risk scores, only a small number of these have been developed further into practical tools that can be used in the clinic or population level screening. That is, the questions or questionnaires that assess the risk factors have been published and made available along with a scoring system. These tools can also be converted into apps and websites [e.g., (14, 42)] and used in clinical practice (see Figure 1).

### Risk Score Implementation

Once a risk score has been developed and validated, it can be used in various ways across a range of settings including population health, research, clinics, and personalized medicine.

#### Population Health

Risk scores can be used to analyze, track, and evaluate the health of populations over time [see (43, 44) for examples]. They may direct policy and guide the allocation of resources for health services and programs. They may also be used to model the economic impacts of dementia and cognitive decline at a state or national level, or to identify geographic hotspots where there are high numbers of individuals with high levels of dementia risk (45).

#### Research

Risk scores can be useful in a research context to: (a) select individuals (e.g., high risk individuals, or individuals with specific risk profiles) for clinical trials [for example (46)], (b) design effective interventions targeted for specific risk profiles, (c) measure the impact of interventions on dementia risk in real time without having to wait for dementia onset, and (d) tracking trends, and any changes in dementia risk profiles over time in sub-populations or community groups through cohort studies (47).

#### Clinics

At the individual level, when risk scores are converted into risk tools, they can be used by primary care providers, specialists, and allied health to provide health advice for individual patients, or specific at-risk groups (e.g., diabetes, stroke, obesity etc.). They can be incorporated into routine health checks in some public health systems including chronic disease health checks for adults at different ages [e.g., the UK midlife health check (48)]. Private insurers may provide rebates for preventive health assessments. Knowing the risk profile of a patient can also help health professionals recommend targeted interventions and health advice for individuals with specific risk levels or profiles. Additionally, it is beneficial from a patient perspective to know their own level of risk for dementia. Risk scores help provide feedback on the effectiveness of preventive health activities in (close to) real-time and provide a target for patients to work toward (49, 50).

#### Personalized Medicine

Risk tools that include detailed biological and clinical information could be used to inform personalized medicine services for brain health. The use of biomarkers (e.g., genetic, blood and brain markers) can lead to highly specific risk assessments with targeted recommendations including tailored pharmacological and non-pharmacological therapies (51).

## Examples of Dementia Risk Scores and Their Use

In this section, three examples of risk scores based on modifiable risk factors for dementia or AD which have been implemented into clinical trials or practice (52) are presented in the order in which they were developed. Their developments have been described in detail previously (8, 53, 54), and their algorithms are summarized in Table 1.

**Table 1:** Algorithms of the CAIDE, ANU-ADRI, and LIBRA risk scores.

**Risk factors**	**Points calculation**		
	CAIDE*	**ANU-ADRI^†^**	**LIBRA^‡^**
Age (years)	<47 = 0	<65 = 0 (men and women)	Not included
	47–53 = +3	65–70 = +1 (men); +5 (women)	
	>53 = +4	71–75 = +12 (men); +14 (women)	
		76–80 = +18 (men); +21 (women)	
		81–85 = +26 (men); +29 (women)	
		86–90 = +33 (men); +35 (women)	
		>90 = +38 (men); +41 (women)	
Sex	Women = 0	Weighted together with age	Not included
	Men = +1		
Education (years)	≥10 = 0	>11 = 0	Not included
	7–9 = +2	8–11 = +3	
	<7 = +3	<8 = +6	
Hypertension	SBP ≤ 140 mmHg = 0	Not included	No = 0
	SBP > 140 mmHg = +2		Yes = +1.6
Hypercholesterolemia	STC ≤ 6.5 mmol/l = 0	STC ≤ 6.2 mmol/l = 0	No = 0
	STC > 6.5 mmol/l = +2	STC > 6.2 mmol/l = +3	Yes = +1.4
Obesity^a^	No = 0	Normal weight = 0	No = 0
	Yes = +2	Overweight = +2	Yes = +1.6
		Obese = +5	
Physical inactivity	No = 0	Low physical activity = 0	No = 0
	Yes = +1	Medium physical activity = −2	Yes = +1.1
		Higher physical activity = −3	
Diabetes	Not included	No = 0	No = 0
		Yes = +3	Yes = +1.3
Depression	Not included	CES-D <16 = 0	No = 0
		CES-D ≥ 16 = +2	Yes = +2.1
TBI	Not included	No = 0	Not included
		Yes = +4	
Smoking	Not included	Never = 0	No = 0
		Past = +1	Yes = +1.5
		Current = +4	
Moderate consumption	Not included	No = 0	No = 0
alcohol		Yes = −3	Yes = −1.0
Social engagement	Not included	Highest = 0	Not included
		Medium to high = +1	
		Low to medium = +4	
		Lowest = +6	
Cognitive activity	Not included	Lowest = 0	No high cognitive activity = 0
		Medium = −7	High cognitive activity = −3.2
		Highest = −6	
Healthy diet	Not included	<0.25 fish portions/week = 0	No MeDi = 0
		0.25–2 fish portions/week = −3	MeDi = −1.7
		2–4 fish portions/week = −4	
		>4 fish portions/week = −5	
Pesticide exposure	Not included	Never = 0	Not included
		Ever = +2	
Coronary heart disease	Not included	Not included	No = 0
			Yes = +1.0
Renal dysfunction	Not included	Not included	No = 0
			Yes = +1.1

* *CAIDE predicts 20 year dementia risk at midlife*.

† *ANU-ADRI was calculated only for individuals aged 60 or more. Validation studies followed up participants for an average of 3.5–6 years*.

‡ *The original LIBRA study followed up mid-late life participants for up to 16 years*.

a *Normal weight defined as BMI <2 5kg/m^2^; overweight defined as BMI ≥ 25 kg/m^2^ and <30 kg/m^2^; obesity defined as BMI ≥ 30 kg/m^2^*.

*CES-D, center for epidemiologic studies depression scale; MeDi, mediterranean diet; SBP, systolic blood pressure; STC, serum total cholesterol; TBI, traumatic brain injury*.

### CAIDE

The Cardiovascular Risk Factors, Aging, and Incidence of Dementia (CAIDE) score was developed in a Finnish population-based cohort aged 39–64 years. It returns an estimate of 20-year dementia risk based on the individual's midlife risk factors profile (Table 1). The CAIDE score is the most thoroughly investigated dementia prediction score having been externally validated in several cohorts [e.g., for prediction of cognitive decline (55, 56) and dementia (57)] and investigated in the context of imaging [e.g., brain volumes and cortical thickness (58, 59)] and pathology (60) markers of disease. More ongoing studies are further investigating longitudinal associations between CAIDE score and novel biomarkers of disease (61).

The CAIDE score has also been used in clinical trials that tested the efficacy multidomain lifestyle preventive interventions. In the Multidomain Alzheimer's Preventive Trial (MAPT), participants with a higher CAIDE score benefitted from the multidomain intervention in terms of the primary cognitive outcomes (62). The CAIDE risk score was also used to select individuals at increased risk of cognitive decline recruited in the first successful larger-scale multidomain intervention trial in the dementia prevention field (46). Given the short time frame of this type of randomized controlled trial compared to the time required for a full clinical manifestation of dementia, change in the CAIDE risk score has been also proposed, as a surrogate outcome, instead of the incidence of dementia (63–65).

CAIDE score may become particularly important as selection tool and surrogate outcome in the context of global networks such as World-Wide FINGERS (66) that, stemming from the success of the FINGER trial and now including work across about 40 countries, aims to adapt, test, and optimize the FINGER model to reduce risk across the spectrum of dementia through a novel international approach for resource sharing, data harmonization, and joint trial planning.

To enable access to an audience as wide as possible, including healthcare providers, a mobile application of the CAIDE score has also been developed (42). Such a tool can help users to estimate their dementia risk, and can provide suggestions relating to relevant risk factor reduction (42).

### ANU-ADRI

The Australian National University Alzheimer's Disease Risk Index (ANU-ADRI) was developed for use in public health settings and designed so that it could be completed without clinical assessment (53). ANU-ADRI assesses the presence of 11 risk factors and 4 protective factors for AD (Table 1). It has been externally validated several cohort studies (20, 67) to predict AD, dementia and MCI. In the PATH Through Life Study, adults with low risk scores who had MCI were also more likely to revert to normal between assessments (68).

The ANU-ADRI was developed through evidence synthesis. Risk and protective factors for AD for which high quality evidence was available were identified through existing systematic reviews. The odds ratios for each risk factor was then obtained either from existing meta-analyses or calculated from relevant cohort studies. Questionnaire items for the tool were created and defined based on how each risk factor was described in the cohort study assessments from which the relative risks were obtained. The ANU-ADRI may be applied when data are not available on all items in the score or when data on some risk factors is missing [e.g., (20)]. It has been translated into Portuguese (69) and after consultation with end users, a short form version of the ANU-ADRI was been created with single or shortened questions for risk factor assessment (70) and this was validated against the full length version.

The ANU-ADRI was used as a surrogate outcome measure in a multidomain risk reduction trial of middle-aged adults because it was not reasonable to expect significant cognitive change over 6 months in this age-group (71). It was also used as an outcome measure in a pilot multidomain dementia risk reduction trial in older community dwelling adults (72) and a multidomain trial in middle-aged adults in primary care (73). The ANU-ADRI was used as a secondary outcome in a multi-domain trial for participants with subjective cognitive decline or MCI (74) and in an ongoing large primary prevention internet based trial (75). The ANU-ADRI has also been examined in its relation to a genetic risk score for AD (67) and correlation with brain measures (76). Finally, it has been used in geo-spatial modeling of dementia hotspots in Australia, demonstrating how dementia risk scores can be applied in the policy setting (45).

### LIBRA

The selection of risk factors for the “LIfestyle for BRAin Health” (LIBRA) dementia risk tool was developed from a review of the literature and a Delphi consensus study (77). The tool includes only modifiable risk factors and was developed to assess the risk of dementia and prevention potential at midlife. The algorithm for LIBRA was developed from analysis of the Maastricht Aging Study Cohort (54) (Table 1). The LIBRA index has been shown to predict dementia (78, 79), MCI, and cognitive decline (80) at midlife and in late life. However, a lack of association between the risk factors included in the LIBRA index and dementia was reported in the oldest old (81), possibly due to a higher burden of comorbidities, compared to younger age-groups. In the Doetinchem Cohort Study (82), higher LIBRA scores predicted faster decline in verbal memory, cognitive flexibility, and mental speed, over 10 years. Higher LIBRA scores were also associated with increased risk of incident cognitive impairment, with similar effects across gender and educational level. In the English Longitudinal Study of Aging cohort (83), the LIBRA score was associated with an increased risk of dementia and differences in LIBRA score partially mediated socioeconomic differences in dementia risk. The late-life LIBRA score while still predictive for MCI, was associated with risk of dementia only in APOE ε4 non-carriers (79). The LIBRA index was used as the primary outcome in the Innovative Midlife Intervention for Dementia Deterrence (In-MINDD) randomized controlled feasibility trial (84). It has also been studied to assess potential heterogeneity of intervention effects in a *post-hoc* analysis of the Prevention of Dementia by Intensive Vascular Care (preDIVA) trial (85) and in the FINGER trial (86).

### Strengths and Limitations of the Current Risk Scores

Although their algorithms differ, the current risk scores all include a core set of risk factors. They have been extensively validated, in diverse populations and, in some cases, in the same studies (87), as predictive tools for both cognitive decline (55, 56, 88) and dementia (8, 57). Though not very common, studies have also investigated dementia risk scores in association with biomarkers of neurodegeneration (59, 89). Promising evidence on their suitability as surrogate outcomes has been reported in both primary (71, 73) and *post-hoc* (63, 64, 86, 90) analyses of multidomain prevention trials.

Limitations related to the current body of evidence on dementia risk scores include lack of information on their associations with brain pathologies such as amyloid, tau, and vascular disease markers. The risk scores have also been developed and validated for the most part in high income countries, with no risk tool developed specifically for LMIC. A recent study examined the applicability of dementia risk tools in LMICs including China, Cuba, the Dominican Republic, Mexico, Peru, Puerto Rico, and Venezuela (91). These countries were recruited as part of the 10/66 cohort study which examined the prevalence and impact of dementia in older adults aged 65 and over in LMICs (92). It found that the ANU-ADRI, the Brief Dementia Screening Indicator (BDSI) and the Rotterdam Study Basic Dementia Risk Model (BDRM) had acceptable discriminative ability, but that the CAIDE and Study on Aging, Cognitive and Dementia (AgeCoDe) did not extrapolate well to LMIC (91). The authors note, however, that the CAIDE shows good transportability within a middle-aged cohort and the 10/66 cohorts were older with relatively short follow-ups. They concluded that the ANU-ADRI, BDSI and BDRM models could be used in LMIC but all models would benefit from further refinement.

### Future Directions

The landscape of dementia risk reduction and prevention is constantly changing and evidence for new risk factors accumulates at a rapid pace. This means that risk scores need to be flexible and dynamic to accommodate changes in evidence. For example, the risk scores discussed above do not include newly emerged risk factors such as hearing loss and sleep problems (21). Other future direction and refinements include accounting for the competing effects of medication use on cardiovascular conditions and their subsequent dementia risk as the evidence base for this develops over time [e.g., statins, anti-inflammatories, anti-hypertensives (27)]. In addition, biomarkers based on neuroimaging (e.g., identifying dementia pathology) and blood tests (e.g., identifying genetic risk) could be integrated into future risk scores once an accurate dose-response relationship with dementia risk can be established. Although the cost-benefit and feasibility associated with conducting these additional tests and assessments would need to be considered from a practical sense, especially for LMIC.

## From Evidence to Practice

We have described the methodological considerations for converting evidence on risk factors for dementia into risk scores, the environments where risk assessment tools may be used, and given examples of dementia risk assessment tools that are available through websites or apps. It is essential that risk tools are based on the highest quality evidence. This evidence must justify the selection of risk factors for inclusion and the thresholds that indicate risk and the scoring. If the most appropriate thresholds are not used in a risk assessment tool, it may not provide useful information to inform advice (e.g., example the degree to which physical activity needs to be increased to reduce dementia risk).

Dementia risk tools need to be matched to the target population so that the estimates are applicable. For example, a risk score developed on evidence drawn from studies of older cohorts, will be most applicable in other cohorts of older adults but not necessarily as applicable in cohorts of middle-aged adults. Similarly, consideration must be given to cultural differences in lifestyle risk factors such as diet, social engagement, and physical activity. Finally, when used in a clinical setting, it may be appropriate to consider risk indicators or biomarkers indicative of underlying pathology. However, here we have concentrated on validated risk assessment tools that do not require expensive medical tests.

In order to successfully translate, implement and utilize a dementia risk score into practice, the evidence and algorithms need to be adapted into an accessible and useable tool and accompanied by evidence-based feedback. For example, the ANU-ADRI website provides a personalized report summarizing an individual's risk factors and the CAIDE risk tool has been converted into an app that provides guidance for individuals on risk modification (42).

**Figure 2:**
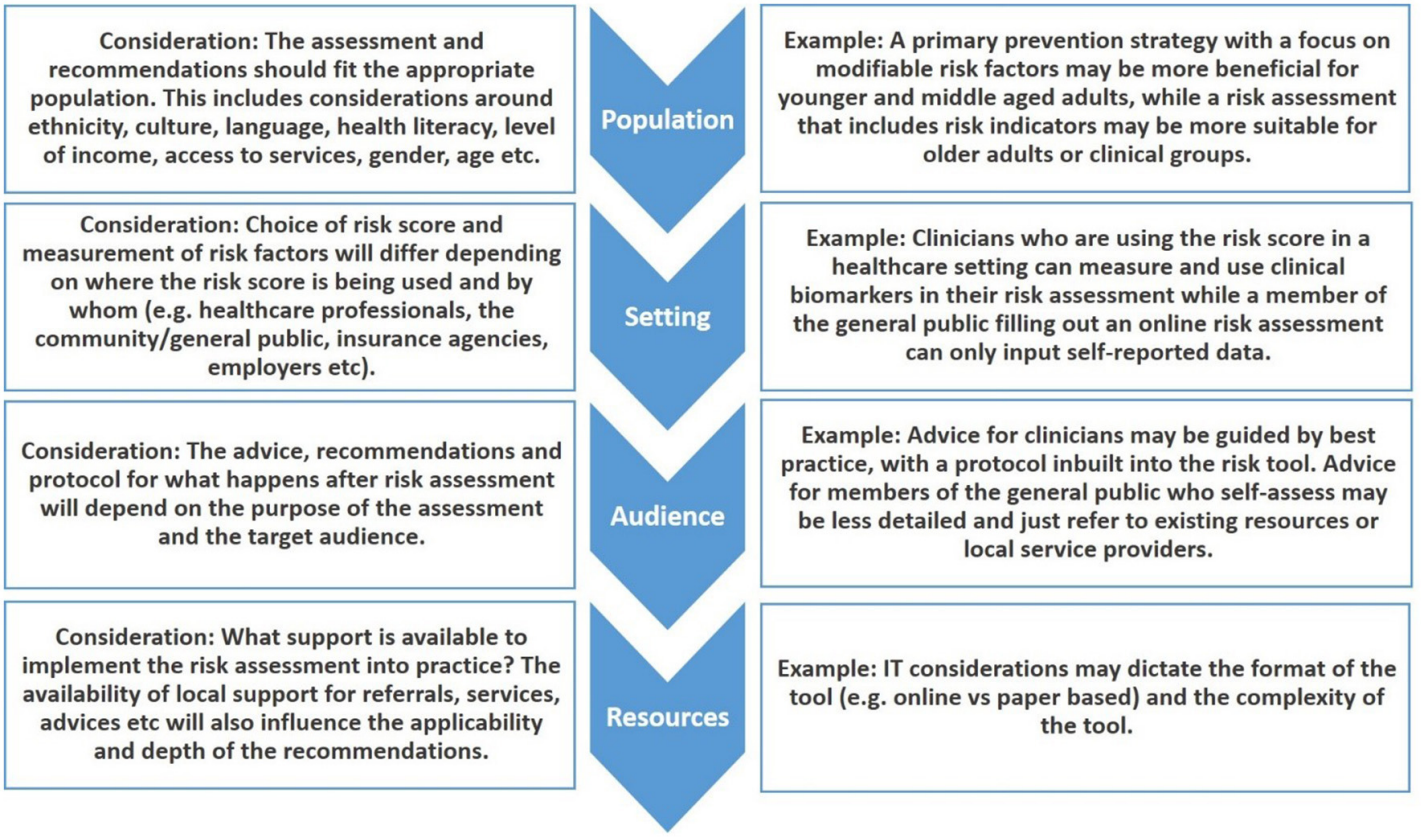
Considerations for selecting or developing risk score for local use.

The format (digital vs. paper based) and relevance of the tool to the local environment are further important factors to consider (93). Ideally, a comprehensive tool provides locally relevant recommendations for the user based on the calculated risk profile, informed by knowledge of available services. Figure 2 provides a summary of the considerations for adapting risk tools for specific situations or environments. Finally, we acknowledge that translation of evidence into practice is a dynamic and iterative process (94) and the implementation of dementia risk tools can be informed by the implementation of other health tools [e.g., guidelines (95)]. This includes assessing barriers to use or uptake; monitoring use over time, evaluating outcomes and effectiveness and facilitating sustained use over time (96, 97).

## Conclusion

Dementia risk tools have been validated in multiple settings and in many populations. Several considerations for the development, use and implementation of dementia risk scores and tools have been detailed in this paper. While further refinement is needed, there is sufficient evidence to use these tools to implement guidelines on dementia risk reduction. At country and regional level, adaptations to local context and evidence-based advice and feedback need to accompany the implementation of dementia risk tools. This will help ensure that dementia risk tools are kept up to date and appropriately prescribed for each population.

## Author Contributions

KA, LZ, RP, and SK contributed to the conception and design of the study. KA wrote the first draft of the manuscript and LZ, RP, SK, MB, RS, AS, and MK wrote sections of the manuscript. All authors contributed to manuscript revision, read, and approved the submitted version.

## Funding

KA and LZ are funded by ARC Fellowship FL190100011. RP and LZ are funded by the NHMRC Dementia Centre for Research Collaboration. LZ is also funded by NHMRC [Grant Number: 1100579]. SK is funded by NHMRC [Grant Number: APP1171279]. MK is funded by Academy of Finland [Grant Numbers: 317465 and 335524], the European Union Joint Programme–Neurodegenerative Disease (JPND) project EURO-FINGERS [Academy of Finland Grant Number: 334804], Stiftelsen Stockholms Sjukhem, Center for Innovative Medicine (CIMED) at Karolinska Institutet, Knut and Alice Wallenberg Foundation, the Swedish Research Council for Health, Working Life and Welfare, Region Stockholm grants (ALF, NSV), and Konung Gustaf V:s och Drottning Victorias Frimurarstiftelse. AS and MB are funded by the European Research Council (ERC) [Grant Number: 804371]. AS and RS are funded by the Finnish Cultural Foundation. AS is also funded by the Academy of Finland [Grant Numbers: 287490 and 319318] and Alzheimerfonden.

## Author Disclaimer

The authors alone are responsible for the views expressed in this article and they do not necessarily represent the views, decisions or policies of the institutions with which they are affiliated.

## Conflict of Interest

KA is an advisor to Staying Sharp. The remaining authors declare that the research was conducted in the absence of any commercial or financial relationships that could be construed as a potential conflict of interest.

## Publisher's Note

All claims expressed in this article are solely those of the authors and do not necessarily represent those of their affiliated organizations, or those of the publisher, the editors and the reviewers. Any product that may be evaluated in this article, or claim that may be made by its manufacturer, is not guaranteed or endorsed by the publisher.
